# Application of Wavelet Transform for PDZ Domain Classification

**DOI:** 10.1371/journal.pone.0122873

**Published:** 2015-04-10

**Authors:** Khaled Daqrouq, Rami Alhmouz, Ahmed Balamesh, Adnan Memic

**Affiliations:** 1 Electrical and Computer Engineering Department, Faculty of Engineering, King Abdulaziz University, Jeddah, 21589, Saudi Arabia; 2 Center of Nanotechnology, King Abdulaziz University, Jeddah, 21589, Saudi Arabia; University of Rome Tor Vergata, ITALY

## Abstract

PDZ domains have been identified as part of an array of signaling proteins that are often unrelated, except for the well-conserved structural PDZ domain they contain. These domains have been linked to many disease processes including common Avian influenza, as well as very rare conditions such as Fraser and Usher syndromes. Historically, based on the interactions and the nature of bonds they form, PDZ domains have most often been classified into one of three classes (class I, class II and others - class III), that is directly dependent on their binding partner. In this study, we report on three unique feature extraction approaches based on the bigram and trigram occurrence and existence rearrangements within the domain's primary amino acid sequences in assisting PDZ domain classification. Wavelet packet transform (WPT) and Shannon entropy denoted by wavelet entropy (WE) feature extraction methods were proposed. Using 115 unique human and mouse PDZ domains, the existence rearrangement approach yielded a high recognition rate (78.34%), which outperformed our occurrence rearrangements based method. The recognition rate was (81.41%) with validation technique. The method reported for PDZ domain classification from primary sequences proved to be an encouraging approach for obtaining consistent classification results. We anticipate that by increasing the database size, we can further improve feature extraction and correct classification.

## Introduction

One of the most common and important protein domains that play an essential role in underlying cell signaling and organizing the post synaptic density region is represented by PDZ domain containing proteins [1–3]. Specifically, PDZ domain proteins have been implicated in functions such as maintainers of cell polarity, regulating the post-synaptic density by mediating protein-protein interactions, and in directing protein trafficking amongst other functions [4–6]. Furthermore, their function, or better yet malfunction, has been characterized in several disease states ranging from cystic fibrosis to cancer [7–9]. PDZ domains primary sequence is usually composed from 80 to 90 amino acids. In addition, most PDZ domains have a conserved 3D fold made of six β strands and two α helices. Almost exclusively, PDZ domains bind the C-terminal motifs of their ligand and target proteins, causing them to cluster [10]. However, occasional internal motifs binding PDZ domains have been observed [11, 12]. The binding pocket of PDZ domains is formed by the conserved GLGF motif, present in most PDZ domains, which usually uses the last four C-terminal amino acids for target recognition. Historically, based on these C-terminal motifs binding the PDZ domain, classification of PDZ domains was proposed. The two most prominent PDZ domain classes are Class I and II. For Class I PDZ domains, a typical motif is S/T-X-Φ, where a hydrophobic amino acid (Φ) is at the C-terminus or position P0, followed by any amino acid (X) at P-1, and then Serine or Threonine at P-2. Alternatively, Class II PDZs would recognize ligand sequence with the Φ-X-Φ- motif at the C-terminus.

In general, PDZ domain classification can be approached from either a structure or sequence based predictions [13–16]. Even though, structural based methods can provide an in-depth understanding of PDZ binding, these methods can be plagued by low throughput and high cost [17–20]. Furthermore, they often provide a glimpse into a single or few PDZ domains, giving less focus to the role of point mutations in PDZ sequence, upstream and downstream sequences relative to the PDZ binding motif, as well as the exact PDZ domain cutoffs that affect ligand affinity [21–23]. More recently, several groups have started work on primary sequence based computational approaches for the determination and prediction of specificity of PDZ domains [18, 24, 25]. However, many of these approaches often group PDZ domains from several species into one category, or even worse, group amino acids into pseudo categories, thus giving less focus on features that are responsible for PDZ domain classifications. Most of the published works are focused on predicting various peptide interactions, with varying levels of success. Two of the most promising works in this field have stemmed from MacBeath lab [25, 26]. This lab initially designed a multidomain selectivity model that implied large promiscuity of many PDZ domain classifications which are instead optimized for selectivity in order to reduce cross-reactivity throughout the mouse proteome. Although the initial correct prediction rate was only 48%, and correct negative prediction for those domains that do not interact at 88%, they built on this model as a precursor for their future work. However, they also focused less on multi species domain structure function relationships and feature extraction techniques. Ultimately, their approach using these [26] previously available results extended the model into a position-specific scoring matrix based on the primary sequence of both the 82 mouse PDZ domains and 93 peptides encoded in the mouse proteome [27]. Another report in the computational analysis using primary sequence data for the classification and prediction of binding interactions of PDZ domains was published by Kalyoncu *et al* [28]. In this work, they approached the challenge from an engineering perspective and focused more on feature vectors and extraction methods. For their study and analysis, they used both bigram and trigram frequency extracted from PDZ domain amino acid primary sequences. Next, they coupled with a random forest classifier for machine learning in order to build their model. Their results were impressive, showing correct classification accuracy at above 90% when using trigrams for classification. They obtained similar results in predicting PDZ domain interactions. To solve the issue with their imbalanced dataset, they used resampling with replacement, which proved to be optimal. However, a major drawback to their study is the grouping of amino acids into categories, diluting how specific amino acid can play a role in PDZ domain classification. To add to PDZ domain classification complexity, Tonikian *et al*. [29] developed a PDZ domain specificity map. They showed that PDZ domain classification can be categorized into 16 unique classes from a database of only 72 PDZ domain members. Furthermore, they were able to introduce the idea of domain mutational effects in the peptide binding prediction. Others like Shao *et al*. describe regression based methods for predicting PDZ domain interactions from primary sequences [30]. It is one of the few methods that try to computationally quantify the strength of peptide-PDZ binding, instead of generating a simple prediction of binding [15]. With this approach, they were able to generate area under curve (AUC) at 0.88 using 23 different PDZ domains. Likewise, Wiedemann *et al*. [31] sought after a quantitative model for three PDZ domains, trying to develop parameters for ligand affinity prediction within a rational design framework of high affinity binders. Similarly, Roberts et al [32] proposed a novel algorithm for predicting binding affinity from complexes of PDZ domains and small peptides.

The purpose of our work is mainly focused on PDZ domain classification aimed at understanding the critical features and sequences motifs responsible for correct classification. In this study, we propose a novel method for PDZ domain classification by using only the primary amino acid sequence information from 115 unique human and mouse PDZ domains. Our goal was to develop novel techniques and approaches based on computational methods that would allow for the best feature extraction responsible for PDZ domain classification relying on natural amino acid sequences and their properties. In specific, the basis of our feature extraction and encoding is represented in the 7 unique physiochemical characteristics of each of the 20 naturally occurring amino acids. Unlike other groups, we avoid using pseudo-amino acid designation in our classification and feature extraction [28]. We have used bigrams and trigrams of the 20 amino acids composing the PDZ domain primary sequences in combination with 7 physiochemical properties of amino acids. We focused on the key elements of occurrence and the existence, as these features are of critical importance to wavelet transformation and later Shannon entropy used in our classification. The reason for this was to determine which of these concepts could be more essential for classification, which we represented through spectrograms. Based on this, we have built our feature extraction methods.

## Materials and Methods

### Dataset

For PDZ domain class classification, we retrieved a total of 115 PDZ domains from human and/or mouse for classification and categorization. The beginning and end of PDZ domain designations were made in accordance with published UniProt annotations, or as they were reported in retrieved datasets [28, 29, 33]. The dataset consisted of 78Class I, and 38 Class II domains. The dataset was based on PDZ domains retrieved from PDZBase [33], and publications by Kalyoncu *et al*. [28], Tonikian *et al*.[29] and Stiffler *et al*. [26]. The full database containing the PDZ domains, their reported classification and primary sequence are provided in the supplementary information as an excel sheet (S1 Data). However, in brief, for the purpose of classification, PDZ domains are denoted as Class I and Class II in accordance with established categorizations; specifically, if the C terminal sequence of the PDZ domain interacting ligand had the [Ser/Thr-X-Φ-COOH] motif, it was denoted as a Class I PDZ domain and if the ligand motif consisted of [Φ-X-Φ-COOH] amino acids at the C-terminus, the PDZ domain was denoted as Class II, respectively. It is important to note that since many high-throughput PDZ screening reports have been published, often a multitude of binders have been identified for a single PDZ domain. Therefore, the basis for inclusion into a specific PDZ domain class was based on the major dominant ligands identified and the motifs they represent, following the classification as mentioned above. Consequently, even if an odd peptide binder not fitting the designed classification had been reported for a given PDZ domain, it was neglected.

### Feature Extraction Procedure

PDZ domains have been characterized as protein scavengers, responsible for protein networking where they are able to reversibly bind multiple protein partners, most commonly through C-terminal interactions [1, 11]. Most empirical methods for determining the specificity and affinity of PDZ-ligand bind pairs are time consuming and expensive, making advanced computational method necessary.

**Fig 1:**
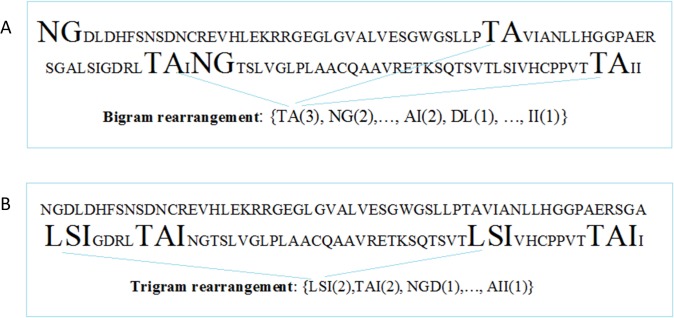
Feature Extraction Approaches. The feature extraction approach by bigram and trigram rearrangements. a) Bigram arrangement, where TA(3) is the pair of amino acids and (3) is the number of repetitions. b) Trigram arrangement, where LSI(2) is the combination of three amino acids and the (2) is the repetition times.

The conserved structure of PDZ domains corresponds to frequency, position and type of amino acids present in the domain fold [1, 11]. In this study, a novel classification of PDZ domains based on primary sequence is proposed. The suggested recognition approach is based on either frequency or existence of two consecutive amino acids (bigram) and three consecutive amino acids (trigram) in the primary sequence. The mentioned combinations can play a crucial role in the way PDZ domains assume their three dimensional fold, thus leading to their function and ligand interaction. Therefore, by identifying the main factors and amino acid combination present in a given primary PDZ domain sequence, we can imply the domain classification. By identifying the driving factors and features behind the PDZ domain structure, we can imply the classification of the individual PDZ domain as it has been previously suggested in studies that correlate differences between Class I and Class II PDZ domains [28]. Using these principles, PDZ domain primary sequences were rearranged based on the following three methods:

1) Occurrence rearrangement method (ORM): this method is based on bigram/trigram occurrence probability in the primary sequence. For instant, '' NGDLD…'' primary sequence will be rearranged as bigrams ''NG'', ''GD'', ''DL'', and ''LD'' or as trigrams ''NGD'', ''GDL'' and ''DLD''. The rearranged PDZ domain sequence will be prioritized by individual bigram/trigram occurrence with those bigrams/trigrams appearing the most given first priority. Next, these bigrams/trigrams will be derived with the corresponding physiochemical characteristics for each amino acid present (Table 1) multiplied by the number of the bi/trigram repetition. These characteristics will be used as the main features for each PDZ domain sequence. Fig 1 illustrates the feature extraction approach by bigram/trigram rearrangements with ORM.

**Table 1:** Physiochemical parameters of the twenty amino acids using the single letter codes.

***Parameter/Amino Acid***	**A**	**G**	**V**	**I**	**L**	**F**	**P**	**Y**	**M**	**T**
**EIIP**	0.0373	0.005	0.0057	0	0	0.0946	0.0198	0.0516	0.0823	0.0941
**Hydrophobicity**	1.8	-0.4	4.2	4.5	3.8	2.8	-1.6	-1.3	1.9	-0.7
**Molecular Weight**	89.09	75.07	117.15	131.17	131.17	165.19	115.13	181.19	149.21	119.12
**Isoelectric point**	6	5.97	5.96	6.02	5.98	5.48	6.3	5.66	5.74	5.66
**Polarity**	8.1	9	5.9	5.2	4.9	5.2	8	6.2	5.7	8.6
**Volume**	31	3	84	111	111	132	32.5	136	105	61
**Composition**	0	0.74	0	0	0	0	0.39	0.2	0	0.71
***Parameter/Amino Acid***	**S**	**H**	**N**	**Q**	**W**	**R**	**K**	**D**	**E**	**C**
**EIIP**	0.0829	0.0242	0.0036	0.0761	0.0548	0.0959	0.0371	0.1263	0.0058	0.0829
**Hydrophobicity**	-0.8	-3.2	-3.5	-3.5	-0.9	-4.5	-3.9	-3.5	-3.5	2.5
**Molecular Weight**	105.09	155.16	132.12	146.15	204.23	174.2	146.19	133.1	147.13	121.16
**Isoelectric poin**	5.68	7.59	5.41	5.65	5.89	10.76	9.74	2.77	3.22	5.05
**Polarity**	9.2	10.4	11.6	10.5	5.4	10.5	11.3	13	12.3	5.5
**Volume**	32	96	56	85	170	124	119	54	83	55
**Composition**	1.42	0.58	1.33	0.89	0.13	0.65	0.33	1.38	0.92	2.75

Numerical values for each of the 7 physiochemical characteristics are mapped to each amino acid and used in the feature extraction and classification methods.

**Fig 2:**
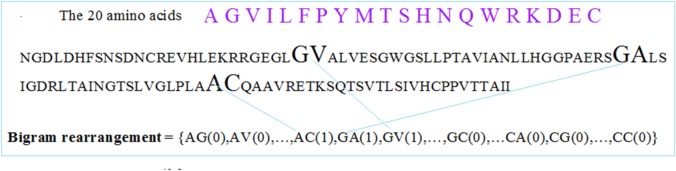
Bigram rearrangement and extraction. Example of bigram by existence rearrangement method on a real primary sequence of PDZ domain.

2) Existence rearrangement method (ERM1) is structured such that the priority in PDZ domain sequence rearrangement is based on the existence or non-existence of an individual bigram/trigram from all possible combinations constructed with the 20 natural amino acids. Therefore, we will screen a single PDZ domain primary sequence for the presence of an individual bi/trigram from all possible combinations, as shown in the Fig 2. We will utilize the same screening protocols using all possible trigram combinations for their presence in a given PDZ domain sequence.

Again, as in the previous method, we substituted each amino acid in the bi/trigram with the seven physicochemical properties [34]. These physiochemical properties included isoelectric points, volumes of side chains, hydrophobicity, solvent accessible surface area, polarizability, and electron-ion interaction potential (EIIP) of amino acids that expresses the average energy states of all valence electrons, as depicted in Table 1.

3) Existence rearrangement method (ERM2) is same to ERM1; however, in this method, we perform an additional step of wavelet decomposition and entropy for each property from the seven physicochemical properties individually, but then combined the result into one vector.

**Fig 3:**
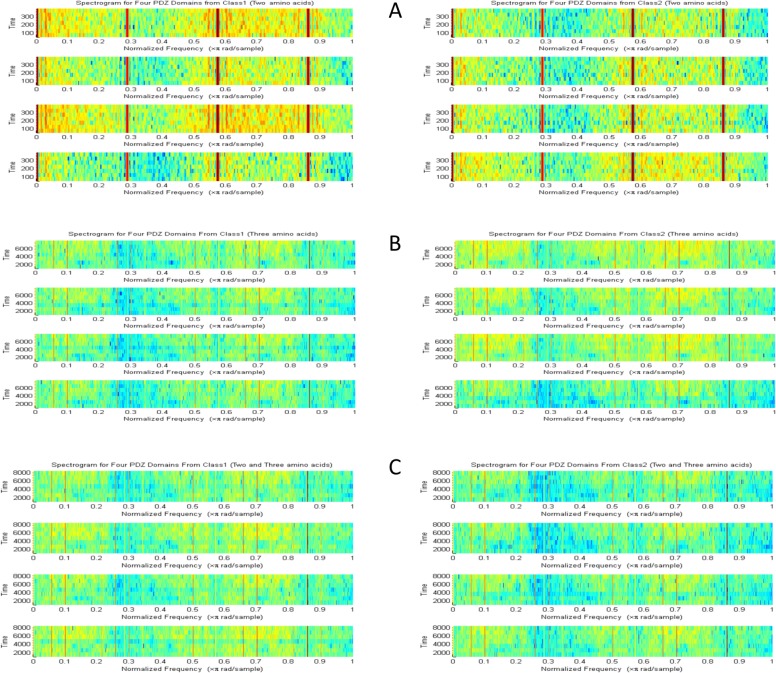
Feature Spectrograms. Spectrogram for bigram and trigram rearrangement features by existence rearrangement method, calculated for four PDZ domains from class1 and different four PDZ from class2. A. Spectrogram for bigram rearrangement features. B. Spectrogram for trigram rearrangement features. C. Spectrogram for fusion bigram and trigram rearrangement features.

In Fig 3, we illustrated spectrograms for bigram and trigram rearrangement features by existence rearrangement method, calculated for four PDZ domains from class1 and different four PDZ from class2. We can notice that features vary widely from class to class, but also have correlation with other features in the same class. However, it is important to correlate back to the distinctive spectrograms fingerprints and feature back to the PDZ domain sequence in order to recognize the key amino acid contributing to PDZ classification.

For better representation of the extracted feature, we use WPT. Wavelet packet transform decomposes the signal into different sub signals of different bands of frequency [35, 36] as:

$$D_{WPT}=\left[c_{1},c_{2},\ldots c_{H}\right]$$ (1)

where *c*_*h*_ is the WPT sub signal, *h* = 1,2,…*H*, where *H* is the number of wavelet packet transform sub signals dependent on the WPT level; in this study, six levels of (J) are used.

In general, entropy is a popular theory utilized in many scientific communities, primarily in the areas of signal and image processing. In the case of classical entropy-based criterion, precise representation for a given image is described by information-related properties. In these entropy-based methods, information related to the concentration of the image is retained, making it popular amongst image processing approaches [37]. Methods measuring entropy are enormously beneficial tools for quantifying the ordering of non-stationary processes. For dimensionality reduction into higher information concentration, Shannon entropy is calculated for each WPT sub signal, as follows:

$$E(s)=-{\sum_{\tau }s}_{\tau }^{2}\text{ log}(s_{\tau }^{2})$$ (2)

where *s* is the input signal, and *s*_i_ are the coefficients of s in an orthonormal basis [38]. The obtained entropy results vector F is the feature vector output:

F=[f1,f2,...,fN] (3)

Here N is the number of elements in the feature vector F which equals to the number of WPT sub signals. Therefore, in the case of bigram ERM2, we decrease the number of features obtained from 2800 to 128.

## Results and Discussion

**Fig 4:**
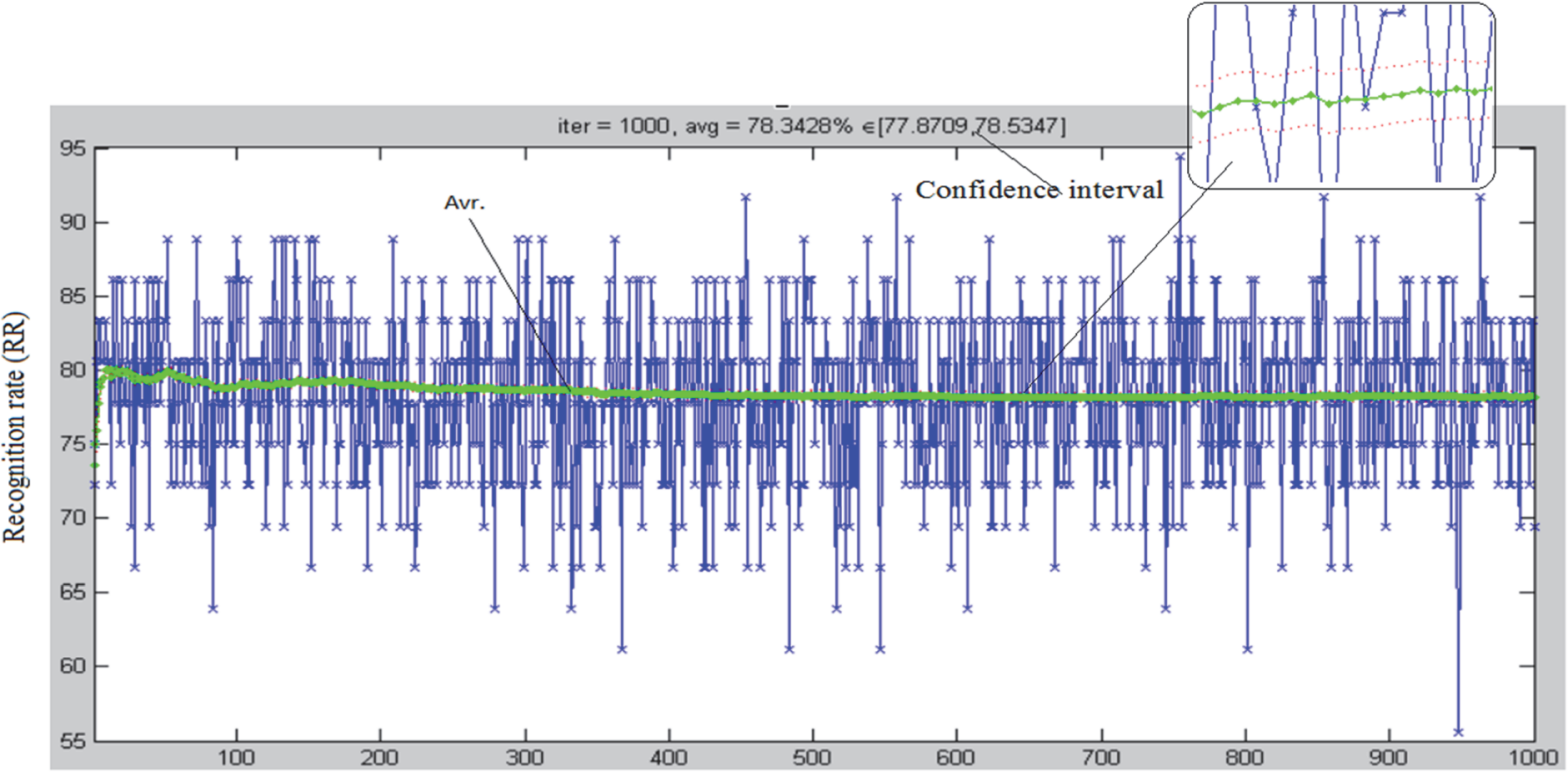
Confidence intervals. Illustration of RR and confidence interval calculated for 1000 different combination training and testing sets.

In order to find out the optimal PDZ domains classification approach, different objective methods are performed. The three suggested feature extraction methods ORM, ERM1, and ERM2 in conjunction with WE (ERWE2) are examined. For PDZ domain classification, support vector machine (SVM), probabilistic neural network (PNN), and K-nearest neighbors (KNN) are utilized. Bigram, trigram and fusion (both of bigram and trigram in the same feature vector) of each PDZ domain primary sequence are investigated. Table 2 shows the results of PDZ domains classification in terms of recognition rate (RR), which is the number of properly classified test samples over the total number of testing samples. The whole database comprising of 115 unique PDZ domains is utilized. We select 57% of PDZ domains from each class for training. Our RR results are calculated as an average of 1000 different combination training and testing sets. In addition, we perform confidence interval measurements representing recognition rates at each step and iteration as depicted in Fig 4. The role of confidence interval is to give an estimated range of possible values including an unknown population parameter, a range estimate for a given set from the sample data. A confidence interval for the recognition rates of the sets combination mean value μ and Standard deviation σ are based on samples size n, therefore,

$$\mu \pm C\left(\sigma /\sqrt{\left(n\right)}\right)$$ (4)

where C is the critical value for a 95% confidence interval (1.96) [39, 40]. Confidence interval results were calculated and reported; specifically, the confidence interval states that 95% of the calculated recognition rate for each combination should be contained in this interval. A wider confidence interval would represent an improper dataset or a database that is unsuitable for performing feature extraction. In our study, all intervals calculated for each method are within a reasonable range.

**Table 2:** Classification Summary.

**Method**		**Bigram**			**Trigram**			**Fusion**		
	RR	BORM	BERM1	BERWE2	TORM	TERM1	TERWE2	FORM	FERM1	FERWE2
SVM	Avr.	69.67	68.65	**74.55**	68.62	69.67	**67.14**	70.01	72.12	**73.89**
	Max.	89	85	**89.81**	88.89	82	**81**	85.23	92	**85**
PNN	Avr.	68.85	70.01	**76.73**	68.62	73.03	**70.91**	68.78	77.01	**72.50**
	Max.	88.89	86	**94.44**	88.89	86	**86.73**	88.89	92	**88.89**
KNN	Avr.	71.31	74.01	**78.34**	65.77	75.59	**76.46**	66.66	76.62	**76.26**
	Max.	94	88.89	**94.44**	86	91.67	**94.44**	81	91.67	**94.44**

Results of PDZ domains classification in terms of recognition rate (RR) Bigram, Trigram and fusion features are presented using three different classifiers.

As observed, the best RR is achieved by means of ERWE2, particularly BERWE2. This is the best feature extraction method, showing the highest RR and using KNN as the PDZ domain classifier. Alternatively, bigram ERM1 (BERM1), trigram ERM1 (TERM1) and fusion ERM1 (FERM1) perform relatively well, making them promising approaches; however, they require further investigation. This leads to a very critical observation related to the feature extraction methods, and the way to make and prioritize bigram or trigram rearrangements. It is clear that the two methods based on the concept of the existence or presence of a given bigram or trigram in the primary sequence is better suited than the method based on occurrences or repetition of each bi/trigram. Furthermore, the results presented in Table 2 demonstrate that ERWE2 outperforms ERM1 in terms of RR, as well as in term of feature vector length; 2800 for **B**ERM1 and 128 for **B**ERWE2.

**Fig 5:**
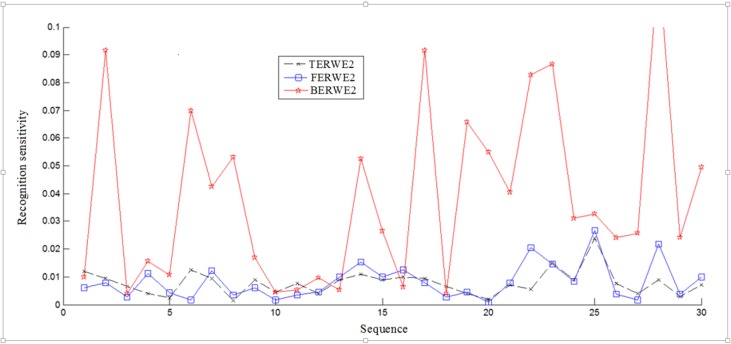
Recognition Sensitivity. The recognition sensitivity results calculated for 30 different PDZ domains.

For more in-depth analysis of the results achieved in Table 2, BERWE2, TERWE2 and FERWE2 are tested and compared further. In order to compare the obtained results, we performed recognition sensitivity (RS) [40] analysis, which was defined as the following:

RS=ρxx−ρxy (5)

where ρ_XX_ is the correlation coefficient determined for two PDZ domain feature vectors belonging to the same class and ρ_XY_ is the correlation coefficient determined for two PDZ domain feature vectors belonging to two different classes. The results representing the recognition sensitivity were determined for 30 combinations of different PDZ domains indicating that these three methods are similar and comparable. However, BERWE2 approach did provide slightly better results. Comparison between the results is illustrated in Fig 5.

Based on the RR results in Table 2 and RS illustrated in Fig 5, we can conclude that the bigram method with ERWE2 is the better when compared to trigrams and fusion methods alone. In general, ERWE2 is shown to be a very promising PDZ domain feature extraction method.

Also examined was the effect of training data quantity on RR results. We use 50%, 57%, 63% and 68% of the database for training and the remaining for testing by means of BERWE2 (data not shown). Results using three classifiers were consistent and dependent on the number of training PDZ domains. KNN approach consistently gave the highest classification percentage.

For more detailed investigation of our results, we applied sensitivity, specificity and positive predictability measures for BERWE2. These measures are defined as following:

$$\begin{array}{l} Sensitivity=TP/\left(TP+FN\right)\\ Specificity=TN/\left(TN+FP\right)\;PositivePredictivity=TP/\left(TP+FP\right) \end{array}$$ (6)

where TP represents the number of true positive samples, which is the number of correctly classified Class1 PDZ domains, where the testing samples were Class1 PDZ domains. FP is the number of false positive samples, which is the number of incorrectly classified Class 1 PDZ domains, where the testing samples were Class1 PDZ domains. TN is the number of true negative samples, which is the number of correctly classified Class 2 PDZ domains, where the testing samples were Class 2 PDZ domains. FN is the number of false negative samples, which is the number of incorrectly classified class2 PDZ domains, where the testing samples were class2 PDZ domains. Table 3 tabulates these three measures, where RR is calculated as the mean value of the three measures.

**Table 3:** Sensitivity measurements.

**Method**	**Sens. (%)**	**Spec. (%)**	**Posit. pred. (%)**	**RR (%)**
**SVM**	84.62	64.29	68.75	72.55
**PNN**	94.69	69.67	71.75	78.69
**KNN**	81.52	76.80	87.50	81.94

Tabulates sensitivity, specificity and positive predictivity measures by BERWE2, where RR is calculated as the mean value of the three measures via 57% training data.

Three published feature extraction methods, amino acid composition (AAC) [41], pseudo amino acid composition (PseAAC) [42] and the auto covariance (AC) method [43], are used for comparison investigation. The results are based on the results published previously by [34]. The comparison is performed by calculating the false positive rate (FPR), true positive rate (TPR), and the accuracy (ACC) rate measuring how well the suggested method performs. Each parameter is defined according to the following relationships: TPR = TP/(FN+TP), FPR = FP/(FP+TN) and ACC = (TP+TN)/(TN +TP+FP+FN). Table 4 tabulates TPR, FPR and ACC, the results of the several feature extraction methods using primary sequence construction that based on different 20 amino acids arrangements. BERWE2 with KNN method is performed by providing 57% of the 115 PDZ domains for training. Although the FPR of the PseAAC (12.28%) is the lowest, its TPR (69.45%) is very low. Overall, the ACC rate (85.19%) obtained by of BERWE2 with KNN method is the highest. However, the TPR (67.95%) obtained by ACC method is the smallest, whereas, TPR (87.50) by BERWE2 with KNN is the best.

**Table 4:** Accuracy with true and false positive results.

**Method**	**No. of features**	**TPR (%)**	**FPR (%)**	**ACC (%)**
**BERWE2 with KNN**	105	87.50	18.18	81.92
**AAC**	40	71.64	15.15	80.24
**PseAAC**	50	69.45	12.28	81.34
**AC**	70	67.95	15.00	79.04

Summary of TPR, FPR and ACC results for several feature extraction methods using primary sequence construction that based on different 20 amino acids arrangements. BERWE2 with KNN method is performed by providing 57% of the 115 PDZ domains for training.

Cross-validation processes the recognition performance in a constant method by excluding a few instances (10% for 10-fold cross-validation) to be used as the test set during the training process [28, 34]. To evaluate our algorithm through another validation technique, we conduct a 10 fold cross-validation test. The test results (with average 81.41) verify that our proposed method is stable and not sensitive to the validation technique.

The proposed method (i.e. BERWE2) was further compared to two more PDZ centric classification methods. The first method is the interaction classification of PDZ domain (ICP) reported by Kalyoncu *et al*. [28]. This method used the frequencies of consecutive trigram and bigram in the primary sequences as features multiplied by the corresponding physiochemical characteristics for each amino acid (Tab.5). The 20 amino acids were clustered into seven different classes according to their dipoles and volumes of the side chains which reflect their interaction specificity. Then for bigram 49, (7×7) features were obtained. The second method is the modified BIRS algorithm (MBIRS) reported by Nakariyakul *et al*.[34], which is a hybrid of the minimal redundancy-maximal-relevance (mRMR) algorithm, and the original best incremental ranked subset (BIRS) algorithm to select relevant and irredundant features for PDZ domain. Then, 215 features were obtained. KNN classifier was used for classification. The classifier was trained by using a 10-fold cross-validation by utilizing our database. The results tabulated in Table 5 show the recognition rates among above two methods compared with BERWE2. The results indicated that the proposed method is superior with a highest recognition rate of 81.41%.

**Table 5:** Comparison with PDZ based methods.

**Method**	**Number of Features**	**Recognition Rate [%]**
BERWE2	128	81.41
ICP	49	71.51
MBIRS	215	74.01

Comparison between two published methods [28, 34] in terms of their recognition rate performed on the dataset used in our current study.

### Critical feature and sequence motifs

**Fig 6:**
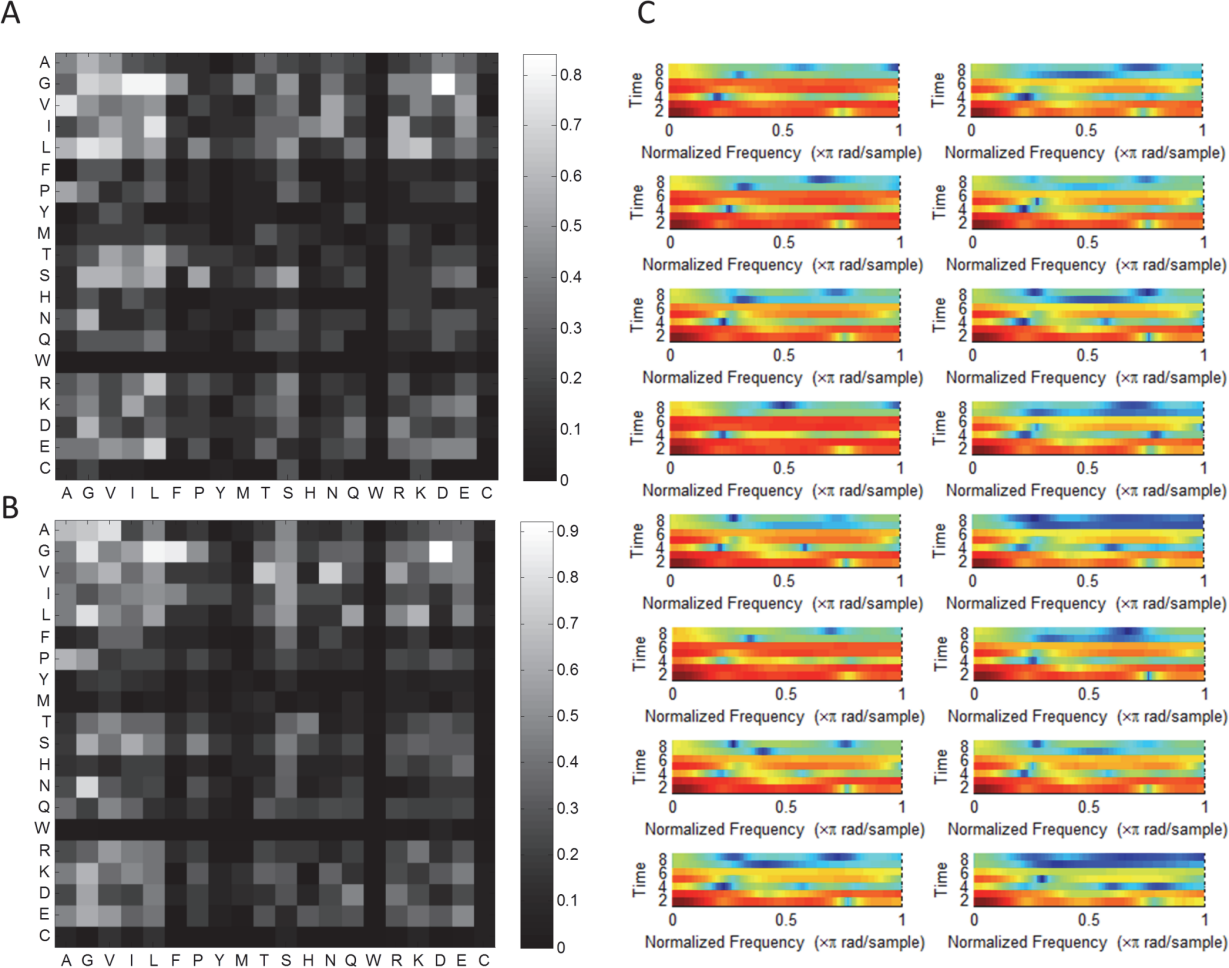
Critical Feature Motifs. The probability of bigrams for A) Class I and B) Class II based on feature extraction in the database (C) Spectrogram for bigram encoded features represented in frequency domain after performing wavelet transformation and Shannon entropy, calculated for unique 8 PDZ domains from Class I (left) and unique eight PDZ from Class II (right).

We wanted to better understand how the selected features help distinguish the two different PDZ domain classes and why they might be important to their specificity. The features extraction method that performed best was based on bigrams (BERWE2). As it was expected, we identify the bigrams that make up the conserved GLGF motif as an important feature. This site is located between βA-βB loop and αB helix and has been well established as one mode of peptide binding and selectivity for all PDZ domains. In order to identify other important motifs, such as the GLGF, we assessed the probability of each bigram appearance. We can see in Fig 6A and 6B a heat map representing the probability of each of bigram from all of the 400 possible combinations. Our feature extraction points to the high probability of bigrams features such as ‘G-Φ’ (stemming from the GLGF motif) and ‘Φ-Φ’ and in particular bigrams of IL, GL, GI, and GD for PDZ domains. Additionally, for Class I PDZ domains, it was also noted that the ‘TH’ bigram has a 48% probability of existence relative to Class II PDZ domains. This is expected as the Histidine often makes hydrogen bonding contacts with Serine or Threonine in position P-2 of the ligands of Class I PDZ domains. Additionally, the appearance of the LG, LQ and LK bigrams for Class II PDZ domains is noteworthy. We can see that these features are propagated and can be visualized with spectrograms (Fig 6C) representative of Shannon entropy of WTP composed of the feature vector based on the seven physiochemical characteristics of amino acids. Even though, positional information is hidden in the spectrograms of Fig 6C we start notice some broad similarities for sequences belonging to Class I (6C left) versus Class II (6C right) PDZ domains.

**Fig 7:**
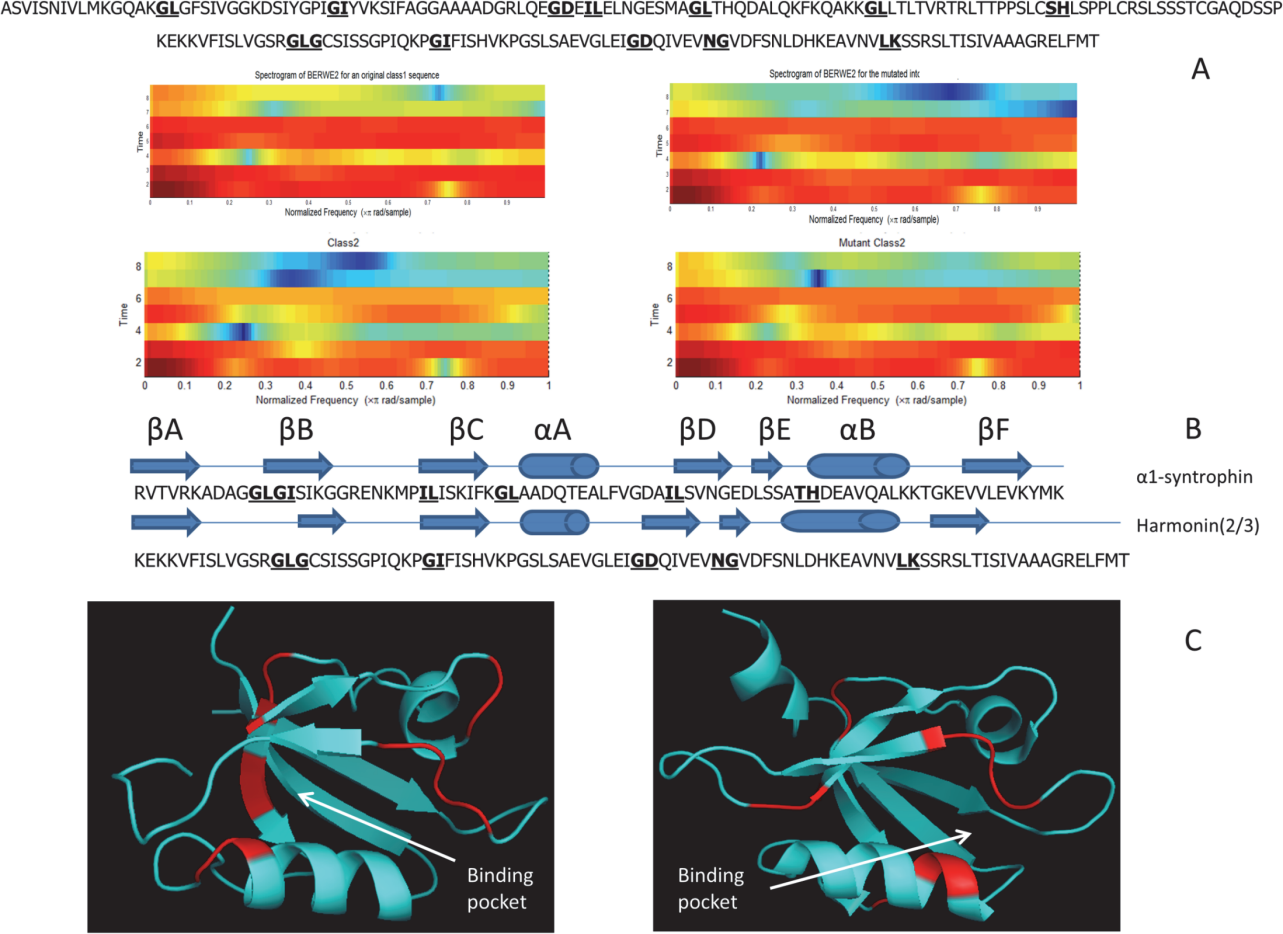
Sequence Motifs. (A) On top is the primary amino acid sequence of pro-interlukin-16 PDZ domain and Harmonin(2/3). Shown in bold and underlined are bigrams that have been mutated. Below is the wavelet spectrograms representative of the original and mutated sequence. (B) The secondary structure positions of the PDZ sequences are represented graphically at the top (αA, Αb, βA-βF) and the primary sequence on the bottom with mutated bigrams shown in bold. (C) Cartoon diagrams of the α1-syntrophin(1/1) (PDB ID:2pdz), and Harmonin(2/3) (PDB ID:2kbs), colored in red are the mutate bigrams showing their relative position to the binding pocket.

In our approach, the benefit of Shannon entropy of WTP is that it helps us perform feature selection leading to dimensional reduction, which in our case is down to 128 features. We hypothesized that if these determined critical feature motifs based on Fig 6 are indeed critical for classification, their effect would be more evident due to the reduction of feature space. We aimed to visualize these differences aided by spectrograms of Shannon entropy of WTP in frequency domain, as a result of mutating the critical feature motifs. In Fig 7A, the top panel represents two primary amino acids sequences (one for a Class I and one Class II PDZ domain) shown with bolded bigrams where manual mutations were performed (making the least pronounced changes such as G→A and I→L). As it can be observed in the bottom panel of Fig 7A, the spectrograms are significantly different when these mutations are introduced, highlighting their importance. It is almost as if there is a class switching between the two sequences when the mutations are introduced. This demonstrates that the spectrograms can be a used tool to quickly assess effects on PDZ domain classification. In Fig 7B and 7C we highlight some of the critical feature and sequence motifs and how they might play a role in PDZ domain interactions. Specifically, we looked at α1-syntrophin PDZ as an example of a Class I PDZ domain. We wanted to map where on the 3D structure the bigrams that we identified as critical motifs are located and correlate this to the published function of the PDZ domain. It was previously reported [44] that LG and GI bigrams corresponding to positions 12–14 in the present sequence undergo a chemical shift with peptide binding. Furthermore, these residues along with Ile15 and Leu68 comprise the binding pocket for the C-terminus of the peptide. In addition, the “TH” bigram is involved in hydrogen bonding with peptide ligands, providing additional recognition specificity. These important motifs are highlighted in red in the 3D structural rendering in the left panel of Fig 7C. Similarly, in the right panel of Fig 7C, the structure of PDZ2 of Harmonin is shown with the critical motifs highlighted in red. As was previously published [45], the GL and LG bigrams that comprise the binding pocket and the GLGF motif are key to ligand recognition, which we determined. Additionally, for Class II PDZ domains LG, LQ and LK, motifs are also critical. The last bigram LK corresponding to the position 71 and 72 in the present sequence are also involved in the stabilization of the ligand pocket and important to PDZ domain classification. However, our approach also highlights other bigrams that don’t appear directly in the binding pocket. We hypothesize that these could be long-range networks [22] that could play a role in PDZ domain folding and structure stabilization, requiring further study beyond the scope of this work.

## Summary

This work studied classification methods of PDZ domain by examining the primary sequences. Three feature extraction methods were proposed. For classification, three known methods were utilized. For better representation of the extracted feature, we used WPT to decompose the signal into different sub signals of different bands of frequency. Shannon entropy was calculated for each WPT sub signal. Thus, we could decrease the number of features obtained BERM1 down to 128. Three methods for feature extraction were tested, one based on the occurrence rearrangement method and the other methods based on the existence rearrangement method. Existence rearrangement was better than occurrence rearrangement in terms of recognition rate. We found that the classification performance of the 115 PDZ domains by ERM2 is better, though not significantly, than those of the two other feature extraction methods. The method ERWE2 based on KNN classifier successfully achieved the recognition rate of 78.34% and the ACC value of 81.92%. For comparison purposes, our proposed method outperformed the three other published methods under similar test parameters. Overall, these types of analyses of sequence space can be done relatively quickly and cheaply in comparison to structure based methods. Specifically, it is important to highlight the time and experimental cost associated with structure based methods allowing for a lesser number of perturbations that can be tested over similar time periods. Although PDZ domains show highly selective interaction pattern, our results indicates with high accuracy that our current classification approach is highly correlated to previously published reports and known classification pattern of PDZ domains. Specifically, our wavelet based approach was able to extract important sequences characteristics and features of PDZ domains. By testing several different methods, we show that the bigram based technique performs the best for classification; from there on, we focused on assessing how these feature can be used to obtain critical motifs. These critical motifs were indeed important and present in the conserved GLGF repeat. Also, we identified the ‘TH’ bigram and specifically Histidine as crucial, which has been shown to make hydrogen bonds with Serine/Threonine of the ligands for Class I domains and for Class II the bigrams of LG, LQ, LK which are often located on αB and also one of the parts of binding pocket. We will concentrate more about the direct concept of position in the future work.

## Supporting Information

S1 Dataset PDZ domain dataset. The first column lists PDZ domain containing proteins given as their common abbreviation. In parenthesis PDZ domain number and sequence in case multiple domains per protein are listed. Next column designates the class of PDZ domain (i.e. either Class 1 or 2). Next column represents the amino acid sequences of the PDZ construct used in our analysis. These boundaries are not necessarily the same as those defined by computational domain identification methods. The domain source (i.e. human or mouse) is designated in the final column.
(XLSX)

## References

[1] Harris BZ, Lim WA (2001) Mechanism and role of PDZ domains in signaling complex assembly. J Cell Sci 114: 3219–3231. 11591811 10.1242/jcs.114.18.3219

[2] Fanning AS, Anderson JM (1999) PDZ domains: fundamental building blocks in the organization of protein complexes at the plasma membrane. J Clin Invest 103: 767–772. 10079096 10.1172/JCI6509 PMC408156

[3] Tsunoda S, Sierralta J, Sun Y, Bodner R, Suzuki E, Becker A et al (1997) A multivalent PDZ-domain protein assembles signalling complexes in a G-protein-coupled cascade. Nature 388: 243–249. 9230432 10.1038/40805

[4] Ataman B, Ashley J, Gorczyca D, Gorczyca M, Mathew D, Wichmann C, et al (2006) Nuclear trafficking of Drosophila Frizzled-2 during synapse development requires the PDZ protein dGRIP. Proc Natl Acad Sci U S A 103: 7841–7846. 16682643 10.1073/pnas.0600387103 PMC1472532

[5] Sans N, Prybylowski K, Petralia RS, Chang K, Wang YX, Racca C, et al (2003) NMDA receptor trafficking through an interaction between PDZ proteins and the exocyst complex. Nat Cell Biol 5: 520–530. 12738960 10.1038/ncb990

[6] Noda Y, Horikawa S, Furukawa T, Hirai K, Katayama Y, Asai T, et al (2004) Aquaporin-2 trafficking is regulated by PDZ-domain containing protein SPA-1. FEBS Lett 568: 139–145. 15196935 10.1016/j.febslet.2004.05.021

[7] Raghuram V, Mak DO, Foskett JK (2001) Regulation of cystic fibrosis transmembrane conductance regulator single-channel gating by bivalent PDZ-domain-mediated interaction. Proc Natl Acad Sci U S A 98: 1300–1305. 11158634 10.1073/pnas.031538898 PMC14749

[8] Ponting CP, Phillips C, Davies KE, Blake DJ (1997) PDZ domains: targeting signalling molecules to sub-membranous sites. Bioessays 19: 469–479. 9204764 10.1002/bies.950190606

[9] Dev KK (2004) Making protein interactions druggable: targeting PDZ domains. Nat Rev Drug Discov 3: 1047–1056. 15573103 10.1038/nrd1578

[10] Doyle DA, Lee A, Lewis J, Kim E, Sheng M, MacKinnon R. (1996) Crystal structures of a complexed and peptide-free membrane protein-binding domain: molecular basis of peptide recognition by PDZ. Cell 85: 1067–1076. 8674113 10.1016/s0092-8674(00)81307-0

[11] Songyang Z, Fanning AS, Fu C, Xu J, Marfatia SM, Chishti AH, et al (1997) Recognition of unique carboxyl-terminal motifs by distinct PDZ domains. Science 275: 73–77. 8974395 10.1126/science.275.5296.73

[12] Sheng M, Sala C (2001) PDZ domains and the organization of supramolecular complexes. Annu Rev Neurosci 24: 1–29. 11283303 10.1146/annurev.neuro.24.1.1

[13] Gujral TS, Karp ES, Chan M, Chang BH, MacBeath G (2013) Family-wide investigation of PDZ domain-mediated protein-protein interactions implicates beta-catenin in maintaining the integrity of tight junctions. Chem Biol 20: 816–827. 10.1016/j.chembiol.2013.04.021 23790492 PMC3728706

[14] Ernst A, Appleton BA, Ivarsson Y, Zhang Y, Gfeller D, Wiesmann C, et al (2014) A structural portrait of the PDZ domain family. J Mol Biol 426: 3509–3519. 10.1016/j.jmb.2014.08.012 25158098

[15] Ernst A, Sazinsky SL, Hui S, Currell B, Dharsee M, Seshagiri S, et al (2009) Rapid evolution of functional complexity in a domain family. Sci Signal 2: ra50. 10.1126/scisignal.2000416 19738200

[16] Ye F, Zhang M (2013) Structures and target recognition modes of PDZ domains: recurring themes and emerging pictures. Biochem J 455: 1–14. 10.1042/BJ20130783 24028161

[17] Jin R, Ma Y, Qin L, Ni Z (2013) Structure-based prediction of domain-peptide binding affinity by dissecting residue interaction profile at complex interface: a case study on CAL PDZ domain. Protein Pept Lett 20: 1018–1028. 23305467 10.2174/0929866511320090008

[18] Hui S, Xing X, Bader GD (2013) Predicting PDZ domain mediated protein interactions from structure. BMC Bioinformatics 14: 27 10.1186/1471-2105-14-27 23336252 PMC3602153

[19] Ernst A, Gfeller D, Kan Z, Seshagiri S, Kim PM, Bader GD, et al (2010) Coevolution of PDZ domain-ligand interactions analyzed by high-throughput phage display and deep sequencing. Mol Biosyst 6: 1782–1790. 10.1039/c0mb00061b 20714644

[20] Mu Y, Cai P, Hu S, Ma S, Gao Y (2014) Characterization of diverse internal binding specificities of PDZ domains by yeast two-hybrid screening of a special peptide library. PLoS One 9: e88286 10.1371/journal.pone.0088286 24505465 PMC3913781

[21] McLaughlin RN Jr., Poelwijk FJ, Raman A, Gosal WS, Ranganathan R (2012) The spatial architecture of protein function and adaptation. Nature 491: 138–142. 10.1038/nature11500 23041932 PMC3991786

[22] Gianni S, Haq SR, Montemiglio LC, Jurgens MC, Engstrom A, Chi CN, et al (2011) Sequence-specific long range networks in PSD-95/discs large/ZO-1 (PDZ) domains tune their binding selectivity. J Biol Chem 286: 27167–27175. 10.1074/jbc.M111.239541 21653701 PMC3149310

[23] Luck K, Charbonnier S, Trave G (2012) The emerging contribution of sequence context to the specificity of protein interactions mediated by PDZ domains. FEBS Lett 586: 2648–2661. 10.1016/j.febslet.2012.03.056 22709956

[24] Hui S, Bader GD (2010) Proteome scanning to predict PDZ domain interactions using support vector machines. BMC Bioinformatics 11: 507 10.1186/1471-2105-11-507 20939902 PMC2967561

[25] Chen JR, Chang BH, Allen JE, Stiffler MA, MacBeath G (2008) Predicting PDZ domain-peptide interactions from primary sequences. Nat Biotechnol 26: 1041–1045. 10.1038/nbt.1489 18711339 PMC2655215

[26] Stiffler MA, Chen JR, Grantcharova VP, Lei Y, Fuchs D, Allen JE, et al (2007) PDZ domain binding selectivity is optimized across the mouse proteome. Science 317: 364–369. 17641200 10.1126/science.1144592 PMC2674608

[27] Hanley JA, McNeil BJ (1982) The meaning and use of the area under a receiver operating characteristic (ROC) curve. Radiology 143: 29–36. 7063747 10.1148/radiology.143.1.7063747

[28] Kalyoncu S, Keskin O, Gursoy A (2010) Interaction prediction and classification of PDZ domains. BMC Bioinformatics 11: 357 10.1186/1471-2105-11-357 20591147 PMC2909223

[29] Tonikian R, Zhang Y, Sazinsky SL, Currell B, Yeh JH, Reva B, et al (2008) A specificity map for the PDZ domain family. PLoS Biol 6: e239 10.1371/journal.pbio.0060239 18828675 PMC2553845

[30] Shao X, Tan CS, Voss C, Li SS, Deng N, Bader GD (2011) A regression framework incorporating quantitative and negative interaction data improves quantitative prediction of PDZ domain-peptide interaction from primary sequence. Bioinformatics 27: 383–390. 10.1093/bioinformatics/btq657 21127034 PMC3031032

[31] Wiedemann U, Boisguerin P, Leben R, Leitner D, Krause G, Moelling K, et al (2004) Quantification of PDZ domain specificity, prediction of ligand affinity and rational design of super-binding peptides. J Mol Biol 343: 703–718. 15465056 10.1016/j.jmb.2004.08.064

[32] Roberts KE, Cushing PR, Boisguerin P, Madden DR, Donald BR (2012) Computational design of a PDZ domain peptide inhibitor that rescues CFTR activity. PLoS Comput Biol 8: e1002477 10.1371/journal.pcbi.1002477 22532795 PMC3330111

[33] Beuming T, Skrabanek L, Niv MY, Mukherjee P, Weinstein H (2005) PDZBase: a protein–protein interaction database for PDZ-domains. Bioinformatics 21: 827–828. 15513994 10.1093/bioinformatics/bti098

[34] Nakariyakul S, Liu ZP, Chen L (2014) A sequence-based computational approach to predicting PDZ domain-peptide interactions. Biochim Biophys Acta 1844: 165–170. 10.1016/j.bbapap.2013.04.008 23608946

[35] Laine A, Fan J (1993) Texture classification by wavelet packet signatures. Pattern Analysis and Machine Intelligence, IEEE Transactions on 15: 1186–1191.

[36] Atal BS (2006) The history of linear prediction. IEEE Signal Processing Magazine 23: 154–161.

[37] Collignon A, Maes F, Delaere D, Vandermeulen D, Suetens P, Marchal G, editors.Automated multi-modality image registration based on information theory; 1995. pp. 263–274.

[38] Coifman RR, Wickerhauser MV (1992) Entropy-based algorithms for best basis selection. Information Theory, IEEE Transactions on 38: 713–718.

[39] Mackowiak PA, Wasserman SS, Levine MM (1992) A critical appraisal of 98.6 F, the upper limit of the normal body temperature, and other legacies of Carl Reinhold August Wunderlich. Jama 268: 1578–1580. 1302471

[40] Daqrouq K, Al Azzawi KY (2013) Arabic vowels recognition based on wavelet average framing linear prediction coding and neural network. Speech Communication 55: 641–652.

[41] Guo Y, Yu L, Wen Z, Li M (2008) Using support vector machine combined with auto covariance to predict protein–protein interactions from protein sequences. Nucleic acids research 36: 3025–3030. 10.1093/nar/gkn159 18390576 PMC2396404

[42] Rashid M, Ramasamy S, Raghava GP (2010) A simple approach for predicting protein-protein interactions. Curr Protein Pept Sci 11: 589–600. 20887258 10.2174/138920310794109120

[43] Chou KC (2001) Prediction of protein cellular attributes using pseudo-amino acid composition. Proteins 43: 246–255. 11288174 10.1002/prot.1035

[44] Schultz J, Hoffmuller U, Krause G, Ashurst J, Macias MJ, Schmieder P, et al (1998) Specific interactions between the syntrophin PDZ domain and voltage-gated sodium channels. Nat Struct Biol 5: 19–24. 9437424 10.1038/nsb0198-19

[45] Pan L, Yan J, Wu L, Zhang M (2009) Assembling stable hair cell tip link complex via multidentate interactions between harmonin and cadherin 23. Proc Natl Acad Sci U S A 106: 5575–5580. 10.1073/pnas.0901819106 19297620 PMC2667052

